# MOBILIZING DOMESTIC FUNDS FOR THE HIV/AIDS RESPONSE IN NIGERIA: ESTIMATING THE POTENTIAL CONTRIBUTION OF THE NATIONAL HEALTH INSURANCE SCHEME

**DOI:** 10.1097/QAI.0000000000003136

**Published:** 2023-04-01

**Authors:** T OLADELE Tolulope, O OLAKUNDE Babayemi, MAO Wenhui, Adekola OLADELE Edward, OGUNDIPE Alex, YAMEY Gavin, OGBUOJI Osondu

**Affiliations:** 1. National Agency for the Control of AIDS, Abuja, Nigeria; 2. Center for Policy Impact in Global Health, Duke Global Health Institute, Duke University, North Carolina, USA; 3. FHI 360, 8 Yedseram Street, Maitama, Abuja, Nigeria; 4. Duke Margolis Center for Health Policy, Duke University, North Carolina, USA; 5. Duke Sanford School of Public Policy, Duke University, North Carolina, USA; 6. Duke University School of Medicine, Department of Population Sciences, Duke University, North Carolina, USA.

**Keywords:** HIV, Resource needs, domestic resources

## Abstract

**Background:**

Amid the dwindling donor support for HIV in Nigeria, there is an urgent need for additional domestic HIV funding. This study estimates the required financial resources for people living with HIV (PLHIV) and the potential magnitude of domestic resources for HIV through the National Health Insurance Scheme (NHIS) and by prioritizing HIV within the health budget.

**Methods:**

We estimated the resource needs for providing antiretroviral therapy (ART) to adults, children, and pregnant women living with HIV under three scenarios: current coverage rates, coverage rates based on historical trends, and a rapid scale-up situation. We conducted a fiscal space analysis to estimate the potential contribution from macroeconomic growth, the NHIS, and prioritizing HIV within the health budget from 2020 to 2025.

**Results:**

At current coverage rates, the annual treatment costs for adults would range between US$ 505 million in 2020 to US$ 655 million in 2025; for children, it ranges from US$ 33.5 million in 2020 to US$ 32 million in 2025. The annual costs of providing PMTCT at current coverage rates range from US$ 65 million in 2020 to US$ 72 million in 2025. An additional US$ 319 million could potentially be generated between 2020 and 2025 through the NHIS for HIV. Prioritizing HIV within the health budget can generate an additional US$ 686 million.

**Conclusion:**

Substantial domestic funds can be mobilized through both means to sustain the HIV response. This additional funding may not be sufficient to cover all PLHIV; a phased approach is recommended.

## Introduction

With about 1.9 million people living with HIV (PLHIV), Nigeria has the second-largest HIV burden in sub-Saharan Africa (SSA)(1). Despite this enormous burden and having one of the largest economies in SSA, the HIV response in Nigeria is mainly donor-dependent. Since 2006, the HIV response has relied on international funding (2,3)with the US President’s Emergency Plan for AIDS Relief (PEPFAR) and Global Fund to Fight AIDS, Tuberculosis and Malaria (GFATM) as the major funders. However, donor funding for HIV has been dwindling in Nigeria (4)due to the global financial crisis, Nigeria’s urge to foster country ownership and its socioeconomic re-designation as a lower-middle-income country.

With the recent impact of COVID-19 on the global economy, development assistance for HIV in Nigeria is unlikely to increase(5). Previous global recessions were associated with an 8–10% drop in official development assistance (ODA) in the first two years post-crisis(6). Within months of the onset of the COVID-19 pandemic, there was a reduction in development assistance, refocusing existing resources on COVID-19; bilateral ODA commitments in the first five months of 2020 were 30% lower than in the same period in 2019. Reducing HIV donor funding resources to Nigeria could jeopardize progress toward ending the HIV/AIDS epidemic as a public health threat by 2030(7).

Facilities introduced user fees to address the dwindling donor funds for HIV and the lack of a proportional rise in domestic funding. A survey of a network of 30 comprehensive HIV treatment facilities in Nigeria showed that 96% of these facilities had introduced user fees to address funding shortages, increasing out-of-pocket (OOP) expenditures among PLHIV(6). PLHIV, especially those living in poor households, are vulnerable to catastrophic health expenditures. OOP for HIV services limit their access to HIV services and perpetuates poverty (2,6,8–10). A study in four states in Nigeria reported that PLHIV spent up to 92% (71%−206%) of their daily income on HIV-related OOP costs (11). Therefore, it is critical for Nigeria to develop sustainable domestic sources to finance its HIV response to prevent backsliding, disease resurgence, and the continued impoverishment of PLHIV.

Earmarking funds for health is essential to increasing the fiscal space for health. Social health insurance can potentially mobilize considerable resources for HIV (12). In Nigeria, the current National Health Insurance Scheme (NHIS) was launched in 2005 to improve access to quality health care, the efficiency of health spending, and financial protection. The scheme is mandatory for all government workers but voluntary for non-government workers. Contributions are related to earnings, with a deduction of 15% of the basic salary entitling an individual, a spouse, and four children below 18 to enjoy comprehensive health benefits. In the face of low enrollment, the NHIS(13) does not support the treatment of chronic conditions such as HIV and cancer to prevent the scheme’s bankruptcy.

In 2014, under the National Health Act, the Nigerian government established the Basic Healthcare Provision Fund (BHCPF) (14). The fund aims to provide a minimum package of healthcare services to Nigerians at the primary healthcare level. It was designed to be resourced by at least 1% of the national Consolidated Revenue Fund (CRF) and contributions from donor agencies and the private sector. The CRF is all receipts by the Federation except those paid into any other public fund established for a specific purpose (15). The private sector will also resource BHCPF. The BHCPF will use three gateways to disburse funds: 45% through the National Primary Healthcare Development Agency (NPHCDA), 50% through the NHIS, offering an opportunity to earmark funds to cover HIV services(14), and the remaining 5% through the Emergency Medical Treatment gateway.

Previous studies showed Nigeria could mobilize additional domestic resources for its HIV response (16–18). With the recently operationalized National Health Act, the grim economic outlook due to COVID-19, and Nigeria’s re-designation to lower-middle-income status, little is known about the potential additional funds for the HIV response. This study estimates the financial resources needed to provide care for PLHIV and the potential magnitude of domestic resources that could be made available for HIV through the NHIS and by prioritizing HIV within the health budget. These estimates should (i) help policymakers put in place appropriate plans for the financial sustainability of the HIV response and (ii) guide other countries that might be transitioning from donor funds.

## Methodology

### Study setting

The study setting is Nigeria, the most populous country in Africa, with a population of about 209 million people(19). HIV prevalence among people 15–49 years is estimated to be 1.3%. Prevalence is higher among women (1.7%) than men (0.8%). It is higher in rural areas (1.4%) than in urban areas (1.1%) (20). Eighty-nine per cent of adults aged 15 years and over living with HIV are on antiretroviral therapy (ART); coverage of pregnant women who receive ART for PMTCT is 44%, while only 45% of children with HIV aged 0–14 years are on treatment(21).

### Analysis

We analyzed four steps: (i) estimation of resources needed to provide ART to all eligible adults, children, and pregnant women between 2020 and 2025; (ii) fiscal space analysis to identify potential additional domestic resources; (iii) estimation of the possible additional domestic resources available to HIV care if HIV was prioritized within the health budget; and (iv) sensitivity analysis.

#### i) Estimation of financial resources needed for HIV care

The financial resources needed were estimated as a function of the number of people living with HIV per year and the annual treatment cost per population group. Table 1 shows the model variables, parameters, and data sources. Estimates of the number of PLHIV in Nigeria from 2020 to 2025 were obtained from the National Spectrum Estimates 2019. Estimates were obtained for three target populations – adults, children, and pregnant women. According to the National Spectrum Estimates, the number of children living with HIV would fall as the years progressed due to more pregnant women accessing ART. ART coverage rates for 2020 were derived from the UNAIDS special analysis 2021, and the UNAIDS epidemiological analysis 2020 served as the baseline. The interventions considered in the computation of an annual treatment cost for HIV were the costs of the drugs for both first and second-line regimens; laboratory services; human resources; the minimal overhead for the facility; and the cost of providing prevention of mother-to-child transmission of HIV/AIDS, including the cost of HIV testing services and ART for a pregnant woman for the duration of her pregnancy.

Annual treatment unit costs for adults and children were derived from the study “Estimating the Cost of HIV Treatment for Adults, Children, and Pregnant Women in Côte d’Ivoire”conducted in 2015 (22). This study considered different age categories and regimen types in arriving at its estimates of annual treatment costs. Studies done in Nigeria did not consider paediatric cases or drug regimen types. Estimates of annual HIV treatment costs for pregnant women were obtained from a Nigerian research due to the vast disparity between this study and the Ivorien study. The annual unit cost for providing an HIV-positive pregnant woman in Nigeria ART services was $US 858, while in Cote D’Ivoire, it was $US 207 (23,24). Excluded were programmatic costs, provision of prevention services, and administrative services at the national level. All costs were adjusted to 2020 US current dollars.

### Coverage scenarios

Three different implementation scenarios were modelled. The first scenario was maintaining the current coverage rate over the medium term. The second scenario was an increase in coverage rate reflective of historical trends and execution capacity. We assumed an average annual rate of change of 3–4% for PMTCT and children. Finally, the third scenario was a rapid scale-up in coverage to attain the UNAIDS 95–95-95 targets in the medium term (95% of PLHIV know their HIV status; 95% of people who know their status are on ART, and 95% of people on ART have suppressed viral loads). The second scenario for adults was not considered due to a high baseline coverage rate of 89%.

#### ii) Fiscal space analysis

We conducted a fiscal space analysis to assess Nigeria’s capacity to provide additional budgetary resources for HIV care without compromising the nation’s financial sustainability and other obligations. We examined the potential impact of macroeconomic conditions on the allocations to the NHIS through the BHCPF and estimated resources potentially generated by prioritizing HIV within the health budget over the medium term (Table 2).

* GDP – Gross Domestic Product, CHE – Current Health Expenditure, GGHE-D – Domestic General Government Healthcare Expenditure, PVT-D – Private Domestic Expenditure, BHCPF – Basic Healthcare Provision Fund. The International Monetary Fund (IMF) GDP growth forecast was used to estimate Nigeria’s real GDP for each year between 2020 and 2025. We assumed that the IMF’s projections of Government revenue as a percentage of GDP in October 2019 were still valid. Based on this, we estimated government revenue for the period 2020 to 2025. These estimates served as a proxy for the CRF from which we estimated the BHCPF from 2020 to 2025.


BHCPF=1%of Government Revenue

NHIS allocation=50%*BHCPF

We then estimated the additional domestic resources that could accrue to the NHIS, including premiums, if current population coverage rates for health insurance were maintained. Premiums were set at a 15% deduction from the median salary in line with the national policy. Copayments were not included as these were not a constant occurrence for every patient visit(13) The additional resources were estimated per annum.

#### iii) Prioritizing HIV within the health budget

The study team estimated the expected resources available to HIV if the current government allocation to HIV as a percentage of the total health budget was maintained. Then we estimated the additional domestic resources available for HIV care if HIV were prioritized within the health budget in line with its contribution to the national burden of disease, as described in equation 1 below. HIV accounts for 7% of Nigeria’s disease burden in disability-adjusted life years (DALYs)(24).


(1) $$\begin{array}{l} Additionalfunds\\ =\sum_{y=0}^{6}\left(GGE_{y}*\frac{GGHED_{y}}{GGE_{y}}*\frac{{\mathrm{HIV DALY}}_{s}}{{\mathrm{Total DALY}}_{s}}\right)\\ -\left(GGE_{y}*\frac{GGHED_{y}}{GGE_{y}}*\frac{\mathrm{HIV Budget}}{\mathrm{Total Health Budget}}\right), \end{array}$$

#### iv) Sensitivity analysis

A probability sensitivity analysis was conducted of the additional domestic resources that could be mobilized using a Monte Carlo simulation to examine two pillars of the fiscal space - allocation to HIV based on its share of the total national burden of disease measured in DALYs and prioritization of the health budget as a percentage of the government budget in line with the Abuja declaration.

## Results

### i) Projected required financial resources needed to provide annual HIV treatment for PLHIVin the medium term

**Figure 1:**
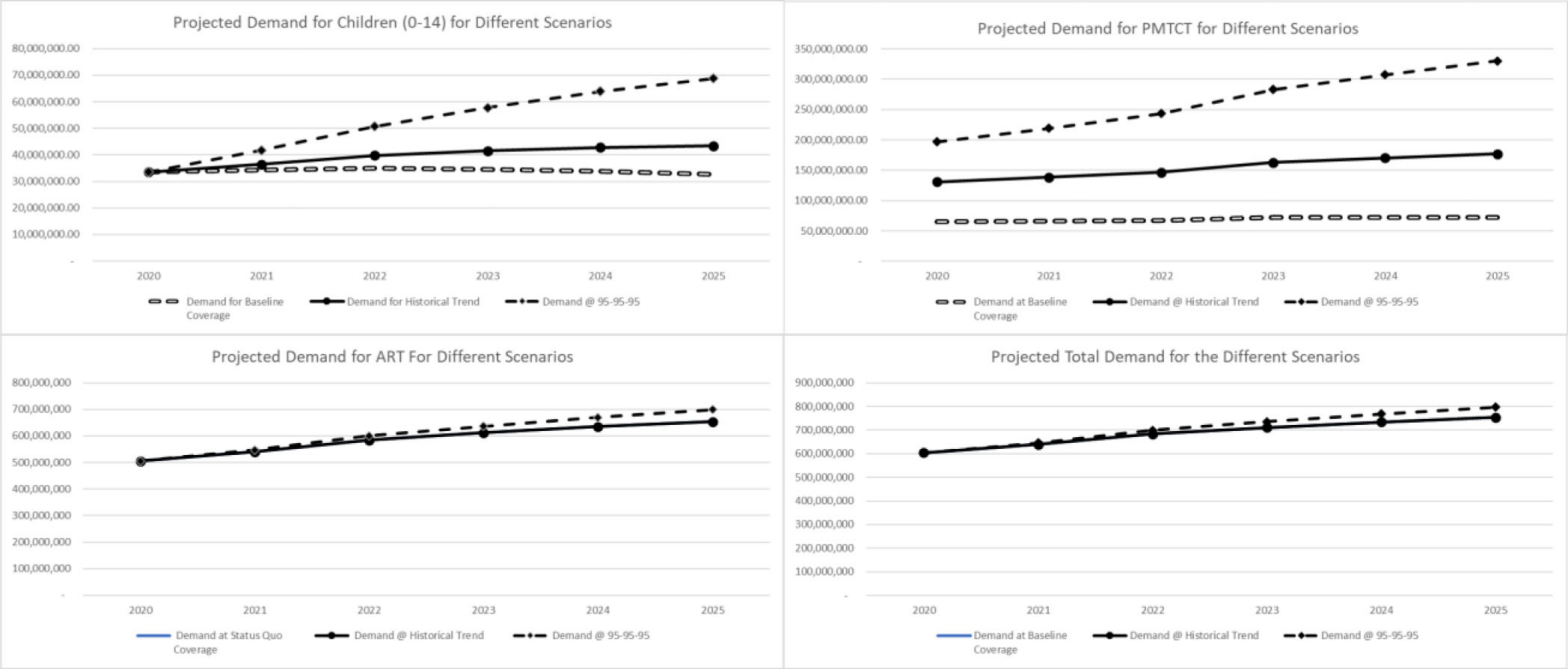
Projected Medium-Term Cost for Different Population Groups Living with HIV

Figure 1 shows the estimated financial requirements for the different target populations at different coverage rates based on the different treatment regimens

If current coverage rates are maintained (baseline) for adults aged 15 years and over, the annual cost of providing ART will range from US$ 505 million in 2020 to US$ 655 million in 2025. To achieve UNAIDS’s rapid scale-up targets of 95–95-95 by 2025, the annual cost of ART provision to adults aged 15 and above would range between US$ 505 million in 2020 and US$ 698 million in 2025.

For children ages 0–14 years, at baseline, maintaining current coverage, the annual treatment cost of HIV would be about US$33.5 million in 2020, falling to US$32.5 million in 2025. ART costs fall because the coverage rate is maintained with a decreasing target population. If coverage rates increase at historical trend rates, the annual cost of ART will range from US$33.5 million in 2020 to US$ 43.4 million in 2025. If the rapid scale-up model is attempted, the annual cost of ART for children will increase to US$ 33.5 million in 2020 and US$ 68.9 million by 2025. The cost of ART in these two scenarios increases due to an increase in percentage coverage of the target population, which overcomes any decrease in the target population.

For pregnant women at baseline, the annual treatment cost would range from US$ 65 million in 2020 to US$ 72 million in 2025. If coverage rates increase at historic trends, the annual cost of ART will increase to US$ 65 million in 2020 and US$ 104 million by 2025. In the rapid coverage scenario, the annual cost of providing ART will increase to US$ 65 million in 2020 and US$ 152 million by 2025.

### ii) Potential domestic resources that could be mobilized

#### Macroeconomic conditions and contributions to the Basic Healthcare Provision Fund

With the impact of COVID-19 on Nigeria’s macroeconomic conditions, the country’s GDP growth rate drops to - 4 percent in 2020 and gradually increase to + 2.5 percent by 2025. This translates to a change from about US$ 440 billion in 2020 to US$ 770 billion in 2025 (Table 2). If historical trends in the ratio of GDP to general government revenues are maintained, revenues will gradually increase from about US$ 26 billion in 2020 to US$ 57 billion in 2025. The macroeconomic conditions model assumes that the GGE (General Government Expenditure) ratio to GDP is 12%. GGE serves as a proxy for CRF (Consolidated Revenue Fund). One percent of the GGE is then allocated to BHCPF, and 50% of the BHCPF funds are earmarked for NHIS. An additional amount from the BPHCF through the state government equity fund is also allocated to NHIS. When GGE is used as a proxy for CRF, and premiums are collected based on the current NHIS coverage rate, a total of US$ 319 million could be raised between 2020 and 2025 through the BHCPF for HIV at an average amount of US$ 53 million annually.

#### Prioritizing HIV within the health budget

If HIV is prioritized within the health budget based on its contributions to the burden of disease in Nigeria in terms of DALYs, a total of US$ 1,247 million could be allocated to HIV compared to a total of US$ 561 million if current allocation trends are maintained (Table 3). This comes to an additional US$ 686 million raised over the next six years.

### iii) Sensitivity analysis

#### Macroeconomic conditions

**Figure 2:**
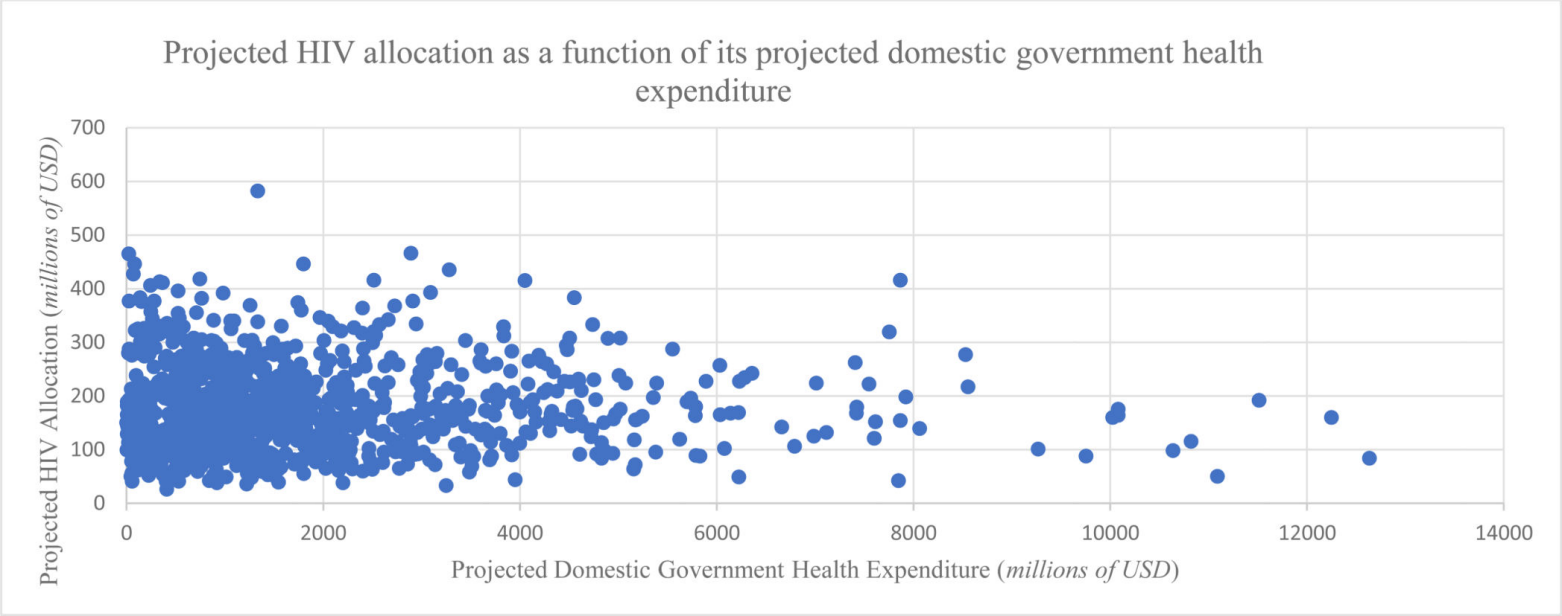
Scatterplot showing the probablistic sensitivity analysis of prioritising HIV allocation within Domestic Government Health Expenditure Notes 1. Assuming HIV receives an equivalent share based on its burden of disease. 2. The evidence suggests that domestic spending will be below 4000 million USD, while HIV spending will hover between 50 to 400 million USD

The mean amount that can be raised in the medium term is US$ 1330 million, with a median of US$ 202.5 million and an interquartile range of US$ 0.005 million – US$ 10,908 million (Table 4 & Figure 2).

#### Prioritizing HIV within the health budget

The mean amount that can be raised in the medium term is US$ 189 million, with a median of US$ 171 million and an interquartile range of US$ 385 million – US$ 2,297 million (Table 4).

## Discussion

Our study shows that if coverage rates are maintained at the status quo, the annual treatment cost for adults aged 15 years and above will range between US$ 505–698million per annum, depending on the scenario. Providing PMTCT for pregnant women will range between US$ 69–108 million per annum, on average, and for children, it will range between US$ 34–53 million per year in the medium term. We estimate that an additional US$ 319–686 million could be mobilized over the next five years by improving macroeconomic conditions or prioritizing HIV within the national health budget.

The resource needs estimated from our study are much lower than estimates from a previous study by Resch et al. (25), which ranged between US$ 0.7 and 1 billion annually from 2012–2018. This difference may be due to the larger population size of PLHIV used in the previous study. Resch et al.’s study was conducted in 2015 based on an HIV prevalence of 3.4%, but the HIV prevalence in Nigeria has since dropped to 1.3% based on the Nigerian HIV/AIDS Indicator and Impact Survey (NAIIS) conducted in 2018 (20)(19)

Consistent with a previous study by the Organisation for Economic Cooperation and Development (22), our study showed that social health insurance is an essential means of mobilizing domestic resources and supports the inclusion of HIV services into the NHIS. With a low coverage of less than 5% and current premium values, it is estimated that a total of US$489 million can be mobilized in the medium term towards HIV care despite the impact of COVID-19 on the economy. This highlights the viability of increased government health spending and health insurance as a vehicle for sustainable funding if appropriately implemented. However, based on our estimates, we recommend integrating HIV care into health insurance for PLHIV should be phased, incorporating different population groups at different times as insurance coverage rates expand. It is important to note that there are ongoing plans to increase the uptake of health insurance among Nigerians through mandatory enrolment for all citizens. (27).

There is uncertainty about how external funding inflows to Nigeria will be shaped in the future. Studies have shown that poor global economic growth is often associated with reduced donor funding (26,27). The advent of the COVID-19 pandemic has led many economies, including high-income countries, to face economic challenges (28,29). The funding landscape of the HIV response in Nigeria seems to be insulated from the shock of the COVID-19 pandemic primarily because of long-term planned investments by its two largest donors - PEPFAR and the Global Fund(30). In June 2020, the Global Fund offered Nigeria a grant of $890 million for the AIDS, tuberculosis, and malaria response for the next three years (2021–2023), the largest funding to any country in the cycle (31). The PEPFAR C0P20 programme aims to increase the percentage of PLHIV who know their status from the current 67% to 81%, increase the percentage of PLHIV on treatment from the current 53% to 76%, and increase the percentage of PLHIV who are virally suppressed from 43% to 72%.

To our knowledge, this study is the first fiscal space analysis for HIV domestic funding since the enactment of the National Health Act in Nigeria. Also, our study uses recent HIV prevalence data from the 2018 NAIIS in arriving at its financial estimates. We were also able to model the impact of the recent COVID-19 pandemic on Nigeria’s macroeconomic conditions.

There are, however, three main limitations to our study. First, our study focused only on three service delivery areas (ART, PMTCT and HIV testing). The cost of other preventive services, including biomedical, structural, or behavioural services, was not included. Also, the cost data were derived from the Ivorien and ORPHTEN studies, which did not include specific cost inputs (these inputs were not components of standard HIV care at the time of both studies but have become part of the standard HIV care). For example, the use of viral load for monitoring viral suppression and the more efficacious antiretrovirals, such as dolutegravir or the fixed combination tenofovir-lamivudine-dolutegravir (TLD), were not captured in both studies. The second limitation is the use of Spectrum estimates in determining resource needs. The Spectrum module uses prevalence and incidence trends and demographic and epidemiological data to produce estimates of indicators of interest, such as the number of people living with HIV, new infections, and AIDS deaths. Due to challenges with empirical measures of HIV prevalence, incidence and mortality, Spectrum estimates are associated with uncertainties. Third, the use of the contribution of HIV to the national burden of disease in DALYs as a guide for resource allocation to HIV through NHIS does not take into consideration other factors, such as equity, political economy, competing priorities etc.

Notwithstanding our study’s limitations, our findings have important policy implications. The first is that adequate funds cannot be mobilized for ART through the BHCPF and NHIS alone. Second, we argue that a phased approach, prioritizing specific populations such as children or pregnant women, is advisable. Our study also makes a case for a vibrant economy mobilizing domestic resources, as in other studies (22–24). Economic growth should be engineered through policies such as government revenue diversification, infrastructure investment, human capital, and effective debt management. Nigeria has the fifth largest population of PLHIV in the world, 1.9 million (32), and adequate plans for financing HIV must be put in place by the government and its partners to prevent the reversal of gains when donors exit or the country loses eligibility for donor funding. Our study highlights the potential role of integration of HIV financing into national health financing, an important issue currently being discussed in many low- and middle-income countries.

## Conclusion

The annual cost of providing ART for PLHIV in Nigeria ranges from US$ 250–500 million, depending on the coverage scenario. Our analysis offers a realistic and optimistic estimate of the fiscal space for HIV, examining two pillars. The results highlight the need for prioritizing funding of HIV in health budgets, prioritizing health in general government expenditures, and strengthening Nigeria’s macroeconomic environment. All three must support sustainable financing of Nigeria’s HIV/AIDS response.

## Figures and Tables

**Table 1:** Model variables, Parameters and Data sources

Model Parameters for estimating Cost			
Indicators	Estimates		Sources
**National Population Projections of People Living with HIV from 2020 to 2025**			
**Population**	**Range of Value**		**Source**
**Pop. LWHIV (15+)**	**2020**	1,712,666	NACA Spectrum Estimates 2019
	**2025**	1,973,750	
**Pop. LWHIV (0–14)**	**2020**	143,025	
	**2025**	116,595	
**Mothers Needing PMTCT**	**2020**	94,694	
	**2025**	95,710	
**Population coverage rates in 2020**			
**Population**	**Value (SD)**		**Source**
Adults	89% (67- >98)		UNAIDS Special Analysis 2021
Children	45% (30–68)		
PMTCT	45% (30–63)		UNAIDS Epidemilogical Estimates 2021
**Variable**		**National Average (Median)**	**Source**
Antiretroviral Treatment for Adults (15+) (Average cost of ARV treatment) 1^st^ Line regimen		**321.7**	
Antiretroviral Treatment for Adults (15+) (Average cost of ARV treatment) 2nd Line regimen		**643.4**	
Prevention of Mother to Child Transmission of HIV/AIDS – 1^st^ line Regimen		**1478.9**	Bautista-Arredondo et al 2018b
Prevention of Mother to Child Transmission of HIV/AIDS −2^nd^ Line Regimen		**2957.8**	
Antiretroviral Treatment for Children (0 – 14) (Average cost of ARV treatment) 1^st^ Line Regimen		**491.4**	
Antiretroviral Treatment for Children (0 – 14) (Average cost of ARV treatment) 2^nd^ Line Regimen		**982.8**	
**Mowdel Parameters for estimating Revenue**			
*Conducive macroeconomic conditions*			
GDP (Millions of US$)		442,976; 466,879; 531,360; 601,920; 680,979; 769,277	World Economic Outlook October 2020
Forecasted Gen. Govt. Revenue as a % of GDP		5.9%,7.1%,7.1%,7.2%,7.4%,7.4%	
Gen. Govt. Revenue		26202; 33,190; 37,907. 43,495; 50,563; 56,603	
BHCPF		1% of Consolidated Revenue Fund	National Health Act 2014
NHIS gateway of BHCPF		50% of BHCPF	
State Equity Fund		25% of BHCPF	
Median salaries		N339,000.00	http://www.salaryexplorer.com
Percentage of basic income taken as premium		15%	NHIS
*Prioritization of HIV within the health budget*			
Gen. Govt. Expenditure		56,059, 56,404, 64,964, 70,118, 81,384, 91,898	World Economic Outlook October 2020
Government spending on HIV as a % of the health budget		0.22% – 4.2%	UNAIDS HIV Financial Dashboard
GGHE-D as % of GGE (2013 – 2018)		4 – 5%	National Health Accounts
Burden of HIV as a % of total burden of disease (Nigeria, Global)		1.88%, 7%, 9.9%	Institute of Health Metrics and Evaluation

**Table 2:** Potential revenue allocations to HIV care from changing macroeconomic conditions

	2020	2021	2022	2023	2024	2025	Total
**Economic conditions and government spending**							
GDP current prices (millions of US$)	442,976	466,879	531,360	601,920	680,979	769,277	3,493,391
General Government Revenue as percent of GDPfrom WEO estimates	6	7	7	7	7	7	42
General Government Revenue (millions of US$)	26,202	33,190	37,907	43,495	50,563	56,603	247,961
**Allocations to HIV/AIDS care and treatment**							
Basic Healthcare Provision Fund (BHCPF) - 1% of Consolidated Revenue Funds in millions of dollars	262	332	379	435	506	566	2,480
BHCPF Appropriation to NHIS - 50% of the BHCPF in millions of US$	131	166	190	217	253	283	1,240
Total State Equity Fund (25% of BHCPF) in Millions of US$	66	83	95	109	126	142	620
Annual Premium Contribution (15% of salary) millions of US$	1,379	1,379	1,379	1,379	1,379	1,379	8,274
Total possible funds available to NHIS in millions of US$	1,575	1,628	1,663	1,705	1,758	1,803	10,314

1. Projections assumed historical trends as well as recent IMF forecasts that account for changes dues to COVID-19.

2. Assuming that fund allocation to HIV from the NHIS aligns the current budgetary allocation to HIV as a proportion of domestic government expenditure on health based on available data

3. Assuming that fund allocation to HIV from the NHIS aligns with the lowest budgetary allocation to HIV as a proportion of domestic government expenditure on health based on available data

4. Assuming that fund allocation to HIV from the NHIS aligns with the highest budgetary allocation to HIV as a proportion of domestic government expenditure on health based on available data

**Table 3:** Potential revenue allocations to HIV care from prioritizing HIV within health budgets

GOVERNMENT EXPENDITURE	2020	2021	2022	2023	2024	2025	Total
Projected general government expenditure as a percentage of GDP culled from WEO 2020 Data	13%	12%	12%	12%	12%	12%	-
Estimated General Government Expenditure based on historical trends (in millions of US$)	56,059	56,404	64,964	70,118	81,384	91,898	420,826
Domestic govt health expenditure as proportion of general government expenditure	4%	4%	4%	4%	4%	4%	-
**Current:** Projected federal government expenditure on HIV at current trends (in millions of US$)^1,2^	75	75	87	94	109	123	561
**Potential:** Projected federal government expenditure on HIV, if HIV is prioritized based on contribution to total DALYS (in millions of US$)	166	167	193	208	241	272	1,247

1. The average federal government expenditure on HIV as a percentage of Domestic General Government expenditure on health is 3.2% over the six-year period between 2020–2025

2. The average rate of Domestic General Government Expenditure on Health as a percentage of General Government Expenditure on health is 4.2% over the six-year period between 2020–2025.

**Table 4:** Summary Table of Probability Sensitivity Analysis for possible outcomes for each year in the Medium Term

**MACROECONOMIC CONDITIONS**							
	**2020**	**2021**	**2022**	**2023**	**2024**	**2025**	**Summary statistics for the medium term**
**Mean**	1180	1283	1355	1332	1513	1337	1333
**Median**	193	192	212	248	219	189	202.5
**2.5 percentile**	0.02	0.01	0.005	0.02	0.03	0.0	0.0054
**97.5 percentile**	8326	9048	10908	10575	10615	10410	10908
**PRIORITIZING HIV**							
	**2020**	**2021**	**2022**	**2023**	**2024**	**2025**	**Summary statistics for the medium term**
**Mean**	225	180	184	179	182	181	189
**Median**	212	172	174	168	170	170	171
**2.5 percentile**	85	59	62	63	58	62	385
**97.5 percentile**	450	351	371	357	390	362	2297

* Figures in millions of USD
